# Full-face aesthetic treatment with onabotulinumtoxin A: Results from a retrospective real world analysis

**DOI:** 10.1016/j.jpra.2025.11.001

**Published:** 2025-11-13

**Authors:** Arash Jalali, Dagné Pupo, Kyu-Ho Yi

**Affiliations:** a One Clinic MD, Vancouver, Canada; b University of British Columbia, Vancouver, Canada; c Dagné Pupo Clinic, Palma de Mallorca, Spain; d You and I Clinic, Seoul, Korea; e Division in Anatomy and Developmental Biology, Department of Oral Biology, Human Identification Research Institute, BK21 FOUR Project, Yonsei University College of Dentistry, 50-1 Yonsei-ro, Seodaemun-gu, Seoul 03722, Republic of Korea

**Keywords:** Botulinum toxin, Full-face treatment, Neuromodulator, Onabotulinumtoxina, Toxin lift, Upper facial lines

## Abstract

**Background:**

Full-face aesthetic treatment with neuromodulators is common in routine practice but not widely analyzed in clinical studies.

**Methods:**

This was a retrospective, two-center chart review evaluating the effectiveness and safety of full-face onabotulinumtoxinA injections for the aesthetic treatment of adult females, all of whom had severe forehead lines at baseline. Patients received a total dose of 114 U: 64 U in the upper face using the on-label treatment pattern for glabellar, crow’s feet, and forehead lines; 20 U per side in the jawline based on the recently proposed “toxin lift” method; and 10 U in the chin.

**Results:**

Thirty-three females were included (mean age: 42.5 ± 7.6 years). Physician-rated forehead line severity improved from “severe” to “mild” or “none” in all patients at 4 weeks, as assessed using the Facial Wrinkle Scale and Forehead Lines Grading Scale. All study participants showed greater facial symmetry, enhanced jawline contour, and high patient satisfaction (FACE-Q Satisfaction with Outcome, 83.9 ± 9.0; Satisfaction with Forehead and Eyebrows, 95.4 ± 5.7). Adverse events were minor and transient.

**Conclusions:**

Full-face onabotulinumtoxinA was effective in reducing facial lines and improving overall symmetry and jawline contour, with high levels of patient satisfaction, and a favorable safety profile.

**Level of Evidence:**

Level III.

## Introduction

Neuromodulator injections are among the most common aesthetic procedures globally.^1^^,^^2^ Their applications have expanded beyond wrinkle reduction to address diverse aesthetic concerns across the face and neck.^3^, ^4^, ^5^, ^6^, ^7^ Recent FDA approval of onabotulinumtoxinA for temporary improvement of platysma bands further illustrates its evolving role.^8^, ^9^, ^10^

The recently proposed “toxin lift” technique aims to enhance midface volume and jawline contour by weakening the caudal pull of the platysma and enhancing the elevator muscles’ counteraction.^11^^,^^12^ However, the combined use of this method with standard upper facial treatments has not been extensively analyzed.^13^, ^14^, ^15^, ^16^, ^17^, ^18^

The current study aimed to evaluate the clinical effectiveness and safety of a comprehensive, full-face neuromodulator protocol that includes upper facial line correction, the toxin lift, and mentalis treatment—all performed within a single session.

## Materials and methods

This was a retrospective, two-center review of charts from adult female patients who had previously received the described full-face treatment protocol, including off-label use, as part of their routine clinical care between September 2023 and October 2024.

The study was conducted in accordance with the ethical standards of the institutional and national research committee and with the 1964 Helsinki Declaration and its later amendments. All patients had provided standard written informed consent for aesthetic neuromodulator treatment prior to their procedure.

### Study design and patients

Patients with severe forehead lines (Forehead Lines Grading Scale ≥3 and Facial Wrinkle Scale = 3) were included. Exclusion criteria were any filler or neuromodulator treatment within the previous 6 months or history of permanent filler or surgery in the treatment area.

### Techniques

Each patient received 114 U of onabotulinumtoxinA:

- 64 U to the upper face (glabella 20 U, crow’s feet 12 U per side, forehead 20 U)
- 40 U to the jawline (20 U per side) using the toxin lift method
- 10 U to the mentalis muscle

**Figure 1:**
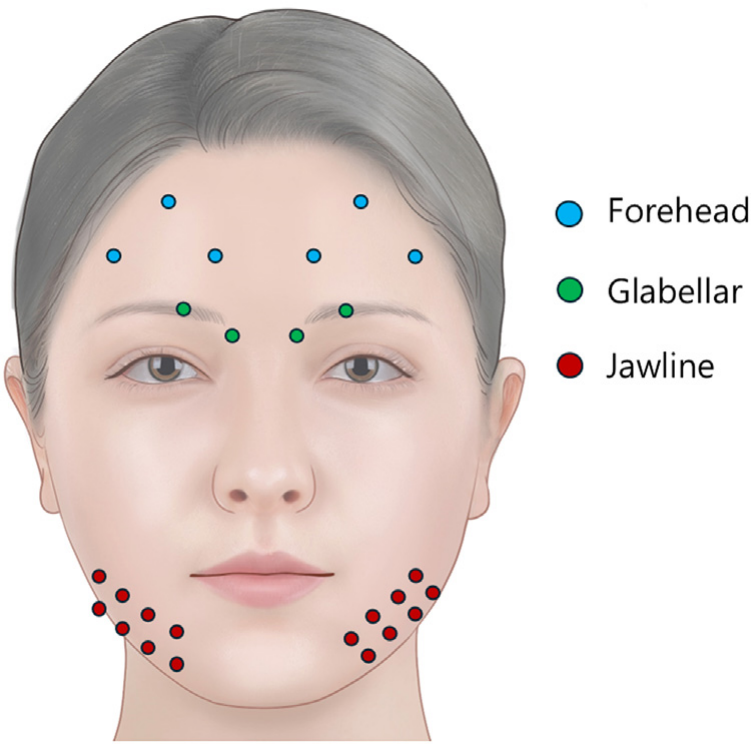
Schematic image of the injection points for the full face neuromodulator. Included patients received 64 U of onabotulinumtoxinA injected into the glabella (20 U), crow’s feet lines (12 U per side), and forehead (20 U). In addition, they were treated with 50 U of onabotulinumtoxinA in the lower face. The majority of this (40 U in total; i.e. 20 U per side) was injected into the jawline using the ‘toxin lift’ method. Blue dot, Forehead injection points; Green dot, Glabellar injection points; Red dot, jawline injection points.

Injection points for the toxin lift were placed 1 cm cranial to the mandibular margin at four locations along the jawline, beginning below the oral commissure and extending toward the mandibular angle (Figure 1).

### Assessments

Physician-rated changes in forehead line severity was assessed at 4 weeks using the Forehead Lines Grading Scale^19^ and the Facial Wrinkle Scale.^20^

Patient satisfaction was measured using FACE-Q Satisfaction with Outcome and FACE-Q Satisfaction with Forehead and Eyebrows instruments.

Adverse events were recorded during treatment and follow-up visits.

## Results

Thirty-three female patients (mean age: 42.5 ± 7.6 years) were included.

Forehead line severity improved from severe (score 3–4) to mild/none (score 0–1) at 4 weeks on both scales.

**Figure 2:**
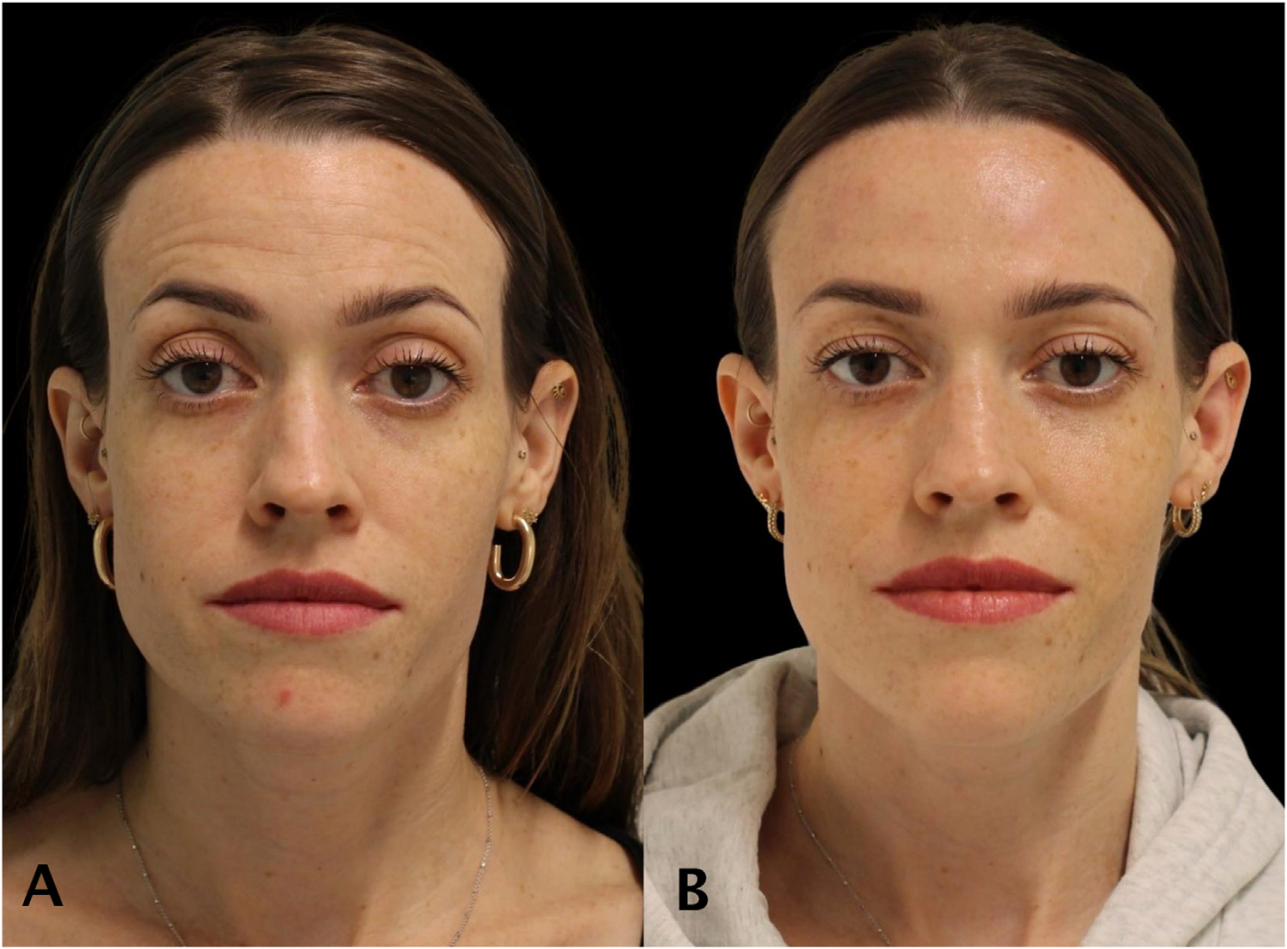
28 year old women treated with full face neuromodulator injections. Forehead line has been dramatically relieved. The patient photo of before (A) and after (B).

**Figure 3:**
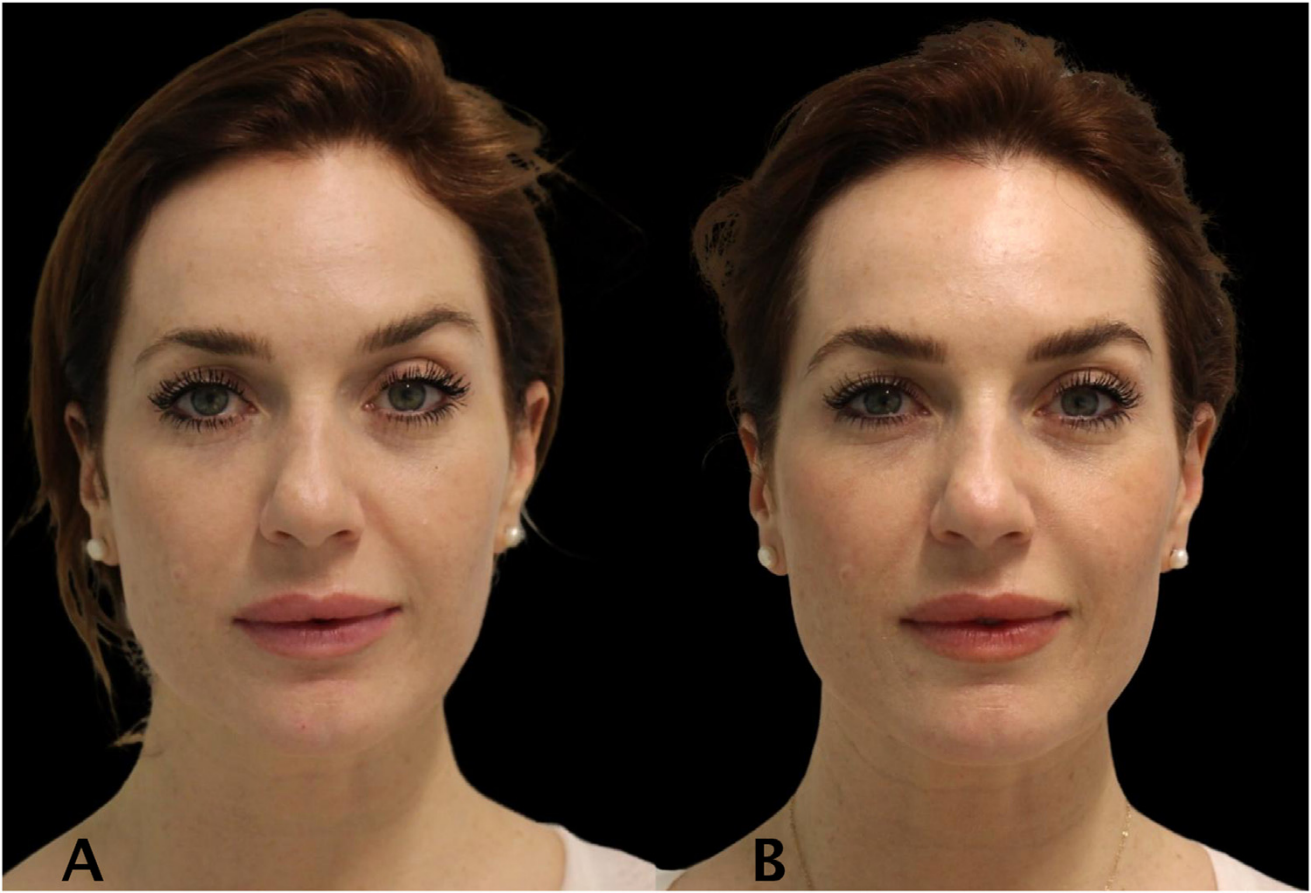
34 year old women treated with full face neuromodulator injections. The lower face has been lifted and reported enhanced overall facial shape and jawline contour. The patient photo of before (A) and after (B).

**Figure 4:**
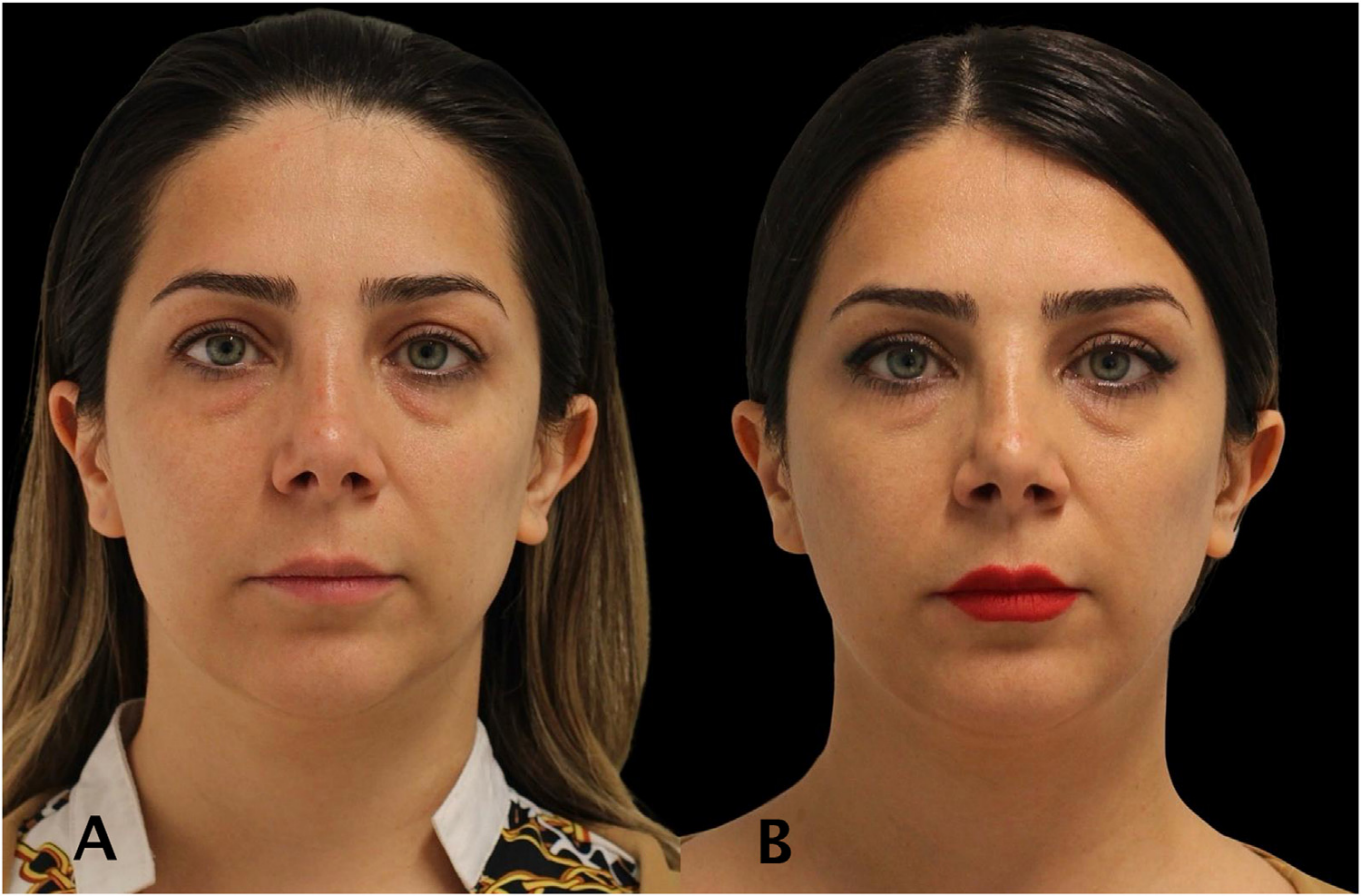
37 year old women treated with full face neuromodulator injections. The lower face and platysma contraction was noted as mild. The patient photo of before (A) and after (B).

**Figure 5:**
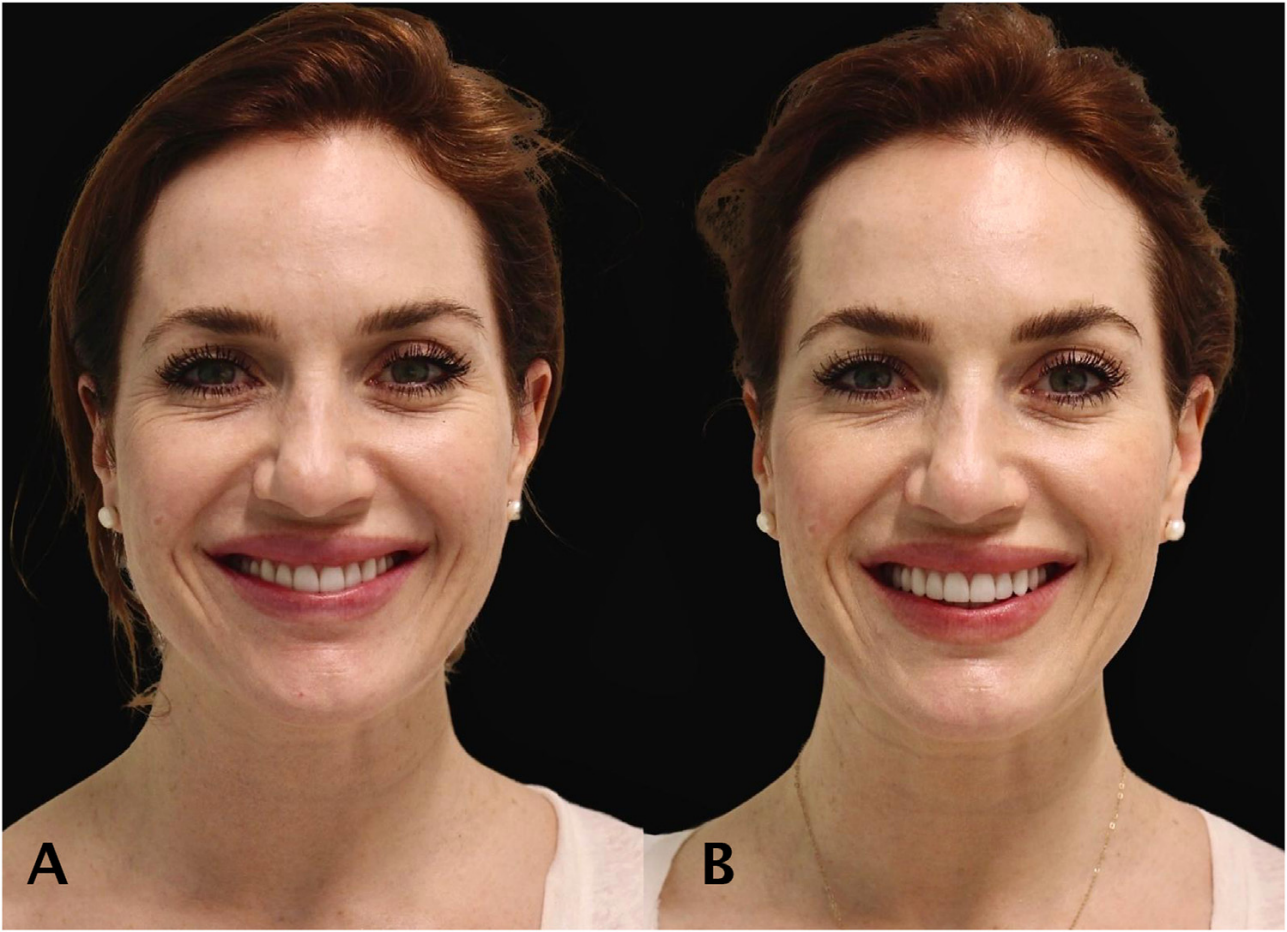
34 year old women treated with full face neuromodulator injections. The patient demonstrated improved symmetry of facial muscle movements during smiling. The patient photo of before (A) and after (B).

Patients demonstrated improved lower-facial symmetry and jawline contour (Figure 2, Figure 3, Figure 4, Figure 5).

Patient satisfaction scores were high:

- FACE-Q Satisfaction with Outcome: 83.9 ± 9.0
- FACE-Q Satisfaction with Forehead and Eyebrows: 95.4 ± 5.7

Adverse events were transient: mild headache (six cases), transient tightness (two cases), and mild, self-resolving dysarthria (one case).

## Discussion

This retrospective, two‑center analysis suggests that a standardized full‑face onabotulinumtoxinA protocol—combining on‑label upper facial treatment with a lower‑face “toxin lift” and mentalis injection—can deliver consistent aesthetic improvements with high patient satisfaction and a favorable safety profile in routine practice. All included women started with severe forehead lines and showed marked improvement to mild or absent lines at 4 weeks on both the Forehead Lines Grading Scale and the Facial Wrinkle Scale. In addition, physicians documented better jawline contour and facial symmetry, and FACE‑Q scores confirmed that patients perceived these changes as meaningful, particularly in the forehead/brow region. Positioning within existing upper‑face literature. These pivotal trials demonstrated high responder rates and durable improvements across multiple upper‑face areas, with predictable onset and duration of action. To our knowledge, few published real‑world series have evaluated a comparable fixed‑dose full‑face pattern with defined jawline injection points and mentalis treatment.^13^, ^14^, ^15^ While the sample size is modest, the consistency of physician‑rated improvement and high FACE‑Q “Satisfaction with Outcome” scores (83.9 ± 9.0) suggest that a structured full‑face approach can be both efficient and reliable, particularly in patients whose concerns include global harmony rather than isolated wrinkles. Jawline contouring and the “toxin lift” The toxin lift concept aims to improve jawline definition and lower‑face contour by weakening the caudal pull of the platysma and related depressor muscles, thereby favoring the elevator vectors of the midface. This principle is grounded in an understanding of facial biomechanics and myomodulation: by selectively reducing downward vectors, the soft tissues of the lower face can appear lifted and more contoured, even without volumizing fillers. Our lower‑face dosing (20 U per side along the mandibular border, 1 cm cranial to the margin, at four points from the oral commissure to the angle) closely follows recently proposed anatomical guidelines for platysma/jawline treatment and facial contouring. Patients in this series demonstrated visibly improved jawline contour and lower‑face symmetry. These observations are consistent with prior anatomical and clinical work demonstrating that carefully placed BoNT‑A injections along the mandibular border can soften platysmal activity, reduce jowl prominence, and sharpen the mandibular outline without compromising function. This may help explain the absence of functionally relevant complications such as dysphagia or significant lower‑face weakness in our cohort, while still allowing visible aesthetic gains. Balancing aesthetic benefit with preservation of natural expression and oral competence remains a key principle in lower‑face neuromodulation. Mentalis hyperactivity can contribute to chin dimpling, labiomental fold prominence, and disharmonious lower‑face movement during speech and smiling. Targeting the mentalis with low‑dose onabotulinumtoxinA is well established and often combined with perioral or jawline injections as part of full‑face rejuvenation.^15^, ^16^, ^17^, ^18^ In our series, inclusion of 10 U to the mentalis in every patient likely contributed to smoother chin contour and improved dynamic symmetry, particularly in the context of jawline repositioning. The clinical photographs illustrate how harmonizing this small muscle can have disproportionate effects on perceived facial balance when integrated into a comprehensive plan. Patient‑reported outcomes and treatment prioritiesRecent survey data underscore that patients’ aesthetic priorities extend beyond isolated wrinkles to include facial harmony, jawline definition, and “looking less tired” or “less sad.” Our FACE‑Q results are consistent with these broader priorities. High satisfaction scores with overall outcome and with forehead/brow appearance suggest that patients value the combined effects on upper‑face expression and lower‑face contour, not just the smoothing of forehead lines. In full‑face neuromodulator strategies, patient‑reported measures such as FACE‑Q complement clinician scales by capturing nuanced aspects of satisfaction, self‑image, and social confidence. Although our study did not include a control group or pre‑treatment FACE‑Q scores for comparison, the absolute values provide useful benchmarks for future prospective studies of similar protocols. Safety in a real‑world full‑face protocol. The safety profile in this cohort was favorable: only minor, transient adverse events (mild headache, transient tightness, and one mild self‑resolving dysarthria) were observed, with no serious complications. This is in line with prior large‑scale clinical trials and real‑world series demonstrating that onabotulinumtoxinA has a wide therapeutic window when injected with correct technique and dosing. Of particular note, no clinically relevant upper‑lid ptosis, eyebrow asymmetry requiring correction, or significant lower‑face functional deficits were recorded. This suggests that the combination of established upper‑face injection patterns and anatomically informed jawline/platysma and mentalis injections can be implemented safely in routine practice when performed by experienced injectors. Nevertheless, the one case of transient dysarthria reminds us that even moderate doses in the lower face can influence perioral function, and careful patient selection, precise anatomy‑based placement, and conservative first‑time dosing remain essential. Several limitations should be acknowledged. First, this was a retrospective chart review without a control group, which limits the ability to attribute all observed changes solely to the treatment and precludes formal hypothesis testing. Selection bias is possible, as only patients with complete baseline and 4‑week follow‑up data were included. Second, the sample size was relatively small (33 women), limiting generalizability and preventing meaningful subgroup analyses by age, facial type, or baseline asymmetry. Male patients, who may require different dosing and patterns because of muscle mass and aesthetic goals, were not represented. Third, all assessments were made at a single time point (4 weeks), close to peak effect for onabotulinumtoxinA. We did not evaluate duration of effect, patterns of wearing off, or the impact of repeated treatments over time. Fourth, physician assessments of symmetry and jawline contour, while clinically relevant, were qualitative; no objective image‑based or three‑dimensional volumetric analyses were performed. Finally, this protocol used a fixed total dose and distribution; individualized dose adjustment based on muscle volume, skin quality, or patient preference—often recommended in consensus guidelines was not explored. Prospective, controlled studies with larger and more diverse populations are warranted to validate these findings, explore dose‑response relationships, and compare this fixed‑pattern full‑face approach with more individualized strategies. Incorporating objective imaging (e.g., standardized three‑dimensional photography or facial movement analysis) could help quantify changes in contour and symmetry more precisely. Longitudinal studies assessing durability, optimal retreatment intervals, and cumulative effects across multiple treatment cycles would also be valuable. Additionally, combining this full‑face neuromodulator protocol with other modalities—such as hyaluronic‑acid fillers, energy‑based devices, or microtoxin techniques for skin quality improvement,—could be studied systematically to develop evidence‑based multimodal algorithms. Finally, future work might examine patient‑reported outcomes across different cultural and age groups, in line with global survey data on aesthetic priorities, to refine full‑face injection strategies that respect both anatomical and cultural aesthetic norms. In summary, this real‑world analysis supports the concept that a standardized full‑face onabotulinumtoxinA protocol, incorporating upper‑face lines, a jawline toxin lift, and mentalis treatment, can safely achieve meaningful improvements in facial lines, contour, and symmetry, with high levels of patient satisfaction. These findings complement existing phase 3 data and consensus recommendations and provide a practical framework for comprehensive neuromodulator‑based facial rejuvenation in daily practice." in the most back.

## Conclusion

Full-face onabotulinumtoxinA treatment effectively improved facial lines, symmetry, and contour with minimal transient side effects, supporting its use as a safe, comprehensive rejuvenation method.

## Funding

None.

## Informed consent

All patients provided written informed consent for aesthetic neuromodulator treatment prior to their procedure.

## Financial disclosure

There is no financial disclosure to report.

## Author contributions

All authors have reviewed and approved the article for submission.

Conceptualization, Arash Jalali

Writing—Original Draft Preparation, Arash Jalali

Writing—Review & Editing, Arash Jalali, Dagné Pupo; Kyuho Yi

Visualization, Arash Jalali, Dagné Pupo; Kyuho Yi

Supervision, Kyu-Ho Yi

## Declaration of competing interest

Arash Jalali has been a speaker and sat on advisory boards for AbbVie, and has been a speaker for Teoxane. Dagné Pupo reports nothing to disclose. Kyu-Ho Yi reports nothing to disclose.

## Appendix Supplementary materials

Image, application 1
