# Supplementary material for: Full-face aesthetic treatment with onabotulinumtoxin A: Results from a retrospective real world analysis
Source: JPRAS Open. 2025 Nov 13;48:223–30. doi: 10.1016/j.jpra.2025.11.001 (PMC12731269; doi:10.1016/j.jpra.2025.11.001)
Supplement: Supplementary file 1 [file mmc1.docx]

Supplement Table 1. Patient satisfaction.

|  | Untransformed sum score (maximum of 24) | Rasch-transformed score (maximum of 100) |
| --- | --- | --- |
| FACE-Q Satisfaction with Outcome | 22.5 ± 1.1 (20-24) | 83.9 ± 9.0 (68-100) |
| FACE-Q Satisfaction with Forehead and Eyebrows | 23.2 ± 1.0 (21-24) | 95.4 ± 5.7 (84-100) |

*Data are given as mean ± standard deviation (range). N=33.
